# β-Cyclodextrin Inclusion Complex to Improve Physicochemical Properties of Pipemidic Acid: Characterization and Bioactivity Evaluation

**DOI:** 10.3390/ijms140713022

**Published:** 2013-06-25

**Authors:** Rosa Iacovino, Filomena Rapuano, Jolanda Valentina Caso, Agostino Russo, Margherita Lavorgna, Chiara Russo, Marina Isidori, Luigi Russo, Gaetano Malgieri, Carla Isernia

**Affiliations:** 1 Department of Environmental, Biological and Pharmaceutical Sciences and Technologies, Second University of Naples, Via A. Vivaldi 43, 81100 Caserta, Italy; E-Mails: valentina.caso@unina2.it (J.V.C.); agostino.russo@unina2.it (A.R.); margherita.lavorgna@unina2.it (M.L.); chiara.russo@unina2.it (C.R.); marina.isidori@unina2.it (M.I.); luigi.russo2@unina2.it (L.R.); gaetano.malgieri@unina2.it (G.M.); carla.isernia@unina2.it (C.I.); 2 Department of Biological and Environmental Science, University of Sannio, Via Port’Arsa 11, 82100 Benevento, Italy; E-Mail: filomena.rapuano@unisannio.it

**Keywords:** β-cyclodextrin, pipemidic acid, inclusion complex, microbial activity, antitumoral activity

## Abstract

The aptitude of cyclodextrins (CDs) to form host-guest complexes has prompted an increase in the development of new drug formulations. In this study, the inclusion complexes of pipemidic acid (HPPA), a therapeutic agent for urinary tract infections, with native β-CD were prepared in solid state by kneading method and confirmed by FT-IR and ^1^H NMR. The inclusion complex formation was also characterized in aqueous solution at different pH via UV-Vis titration and phase solubility studies obtaining the stability constant. The 1:1 stoichiometry was established by a Job plot and the inclusion mechanism was clarified using docking experiments. Finally, the antibacterial activity of HPPA and its inclusion complex was tested on *P. aeruginosa*, *E. coli* and *S. aureus* to determine the respective EC_50s_ and EC_90s_. The results showed that the antibacterial activity of HPPA:β-CD against *E. coli* and *S. aureus* is higher than that of HPPA. Furthermore, HPPA and HPPA:β-CD, tested on human hepatoblastoma HepG2 and MCF-7 cell lines by MTT assay, exhibited, for the first time, antitumor activities, and the complex revealed a higher activity than that of HPPA. The use of β-CD allows an increase in the aqueous solubility of the drug, its bioavailability and then its bioactivity.

## 1. Introduction

**Figure 1:**
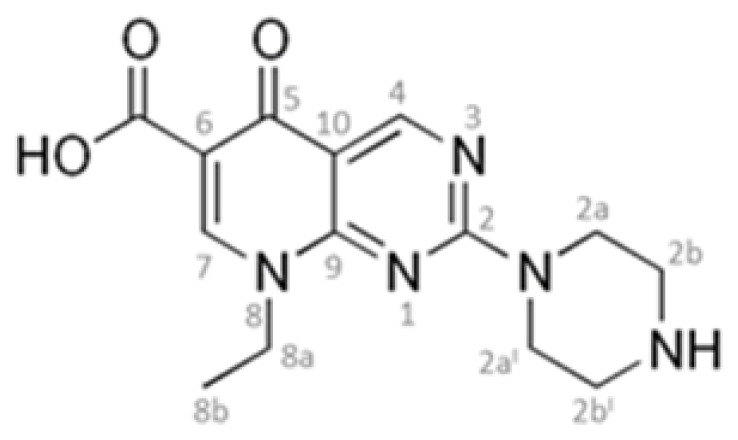
Molecular structure of HPPA.

**Figure 2:**
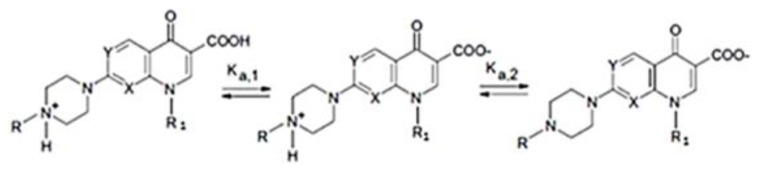
Acid-base equilibrium for piperazinyl quinolones, for HPPA X = Y = N, R = H and R_2_ = CH_2_CH_3_.

Pipemidic acid (HPPA), 8-ethyl-5,8-dihydro-5-oxo-2-(1-piperazinyl)-pyrido[2,3-d]pyrimidine-6- carboxylic acid [1,2] shown in Figure 1, is a therapeutic agent for urinary tract infections because of its antibacterial activity against gram-negative as well as some gram-positive bacteria [3,4]. In the HPPA molecule, a quinolone derivate, the carboxylic group at C6-position makes this compound acidic while the piperazine in position 2 includes an amine group, which is basic. For these reasons, in aqueous solution, 2-piperazinyl quinolone exists in three different species: acidic for pH values under pKa_1_ = 5.4, neutral for pH value closely to isoelectric point (pH = 6.8) and alkaline for pH values higher pKa_2_ = 8.2 [5]. The existing equilibria for these types of quinolones are shown in Figure 2. The HPPA structure is important for its activity; in fact, it shows a better activity against *Pseudomonas aeruginosa* than piromidic and nalidixic acids, structurally related to it [6]. This drug severely damages DNA in the absence of an exogenous metabolizing system and it may act as a multidentate ligand to coordinate with metal ions [7,8]. However, its very low aqueous solubility [9,10] can cause formulation problems and limit its therapeutic application.

Cyclodextrins (CDs) are cyclic oligosaccharides which provide an interesting organic host system, since they have a hydrophobic inner cavity available to form non-covalent host-guest inclusion complexes with a wide variety of organic molecules of appropriate shape and size [11]. CDs are widely used to enhance the aqueous solubility of drugs [12–14] since the physicochemical properties of the guests (solubility, stability, bioavailability, antimicrobial activity, *etc*.) are altered upon complexation. For these reasons, in order to contribute to the increase of the HPPA aqueous solubility and thereby its pharmaceutical applications, we report here the preparation and characterization of inclusion complexes formed by HPPA with native β-CD. The inclusion complex HPPA:β-CD was prepared by kneading method and the adduct was characterized, in the solid state, by Fourier Transform-Infrared spectroscopy (FT-IR) and, in the solution state, by Nuclear Magnetic Resonance (NMR). The stoichiometry and the stability constants (Kb) of the complex, obtained in solution, were determined using the phase solubility diagram (PSD) and Ultraviolet-Visible (UV-Vis) spectroscopy. The influence of the HPPA molecule protonation state on the complex stability was also investigated by estimating the sensitivity of the complex formation constant (Kb) as a function of pH. Furthermore, to characterize the structural details of the complex we carried out a molecular docking study. To evaluate possible differences in antimicrobial efficiency of HPPA compared to HPPA:β-CD complex, different bioactivity tests were performed. The antibacterial activity was tested both on gram-negative and gram-positive bacteria: *Pseudomonas aeruginosa*, *Escherichia coli* and *Staphilococcus aureus*. For each strain, the effective median inhibitory bacterial growth concentration (EC_50_) as well as the respective EC_90_ were determined using the broth-based turbidimetric assay. Previous studies showed that some quinolones exhibited promising cytotoxicity in different human cancer cells [15,16]. For this reason the cytotoxicity of the investigated compounds were tested on human hepatoblastoma HepG2 and human breast cancer MCF-7 cell lines by MTT assay. The inclusion of HPPA with β-CD appears to modulate the capability of the parent compound to penetrate into the cells and improves the antitumor activity of HPPA against breast and hepatocellular cancers, suggesting attractive roles for applications in medicine.

## 2. Results and Discussion

### 2.1. FT-IR Spectroscopy

The FT-IR spectrum gives detailed information about the functional groups involved in the interaction when the complex is formed.

**Figure 3:**
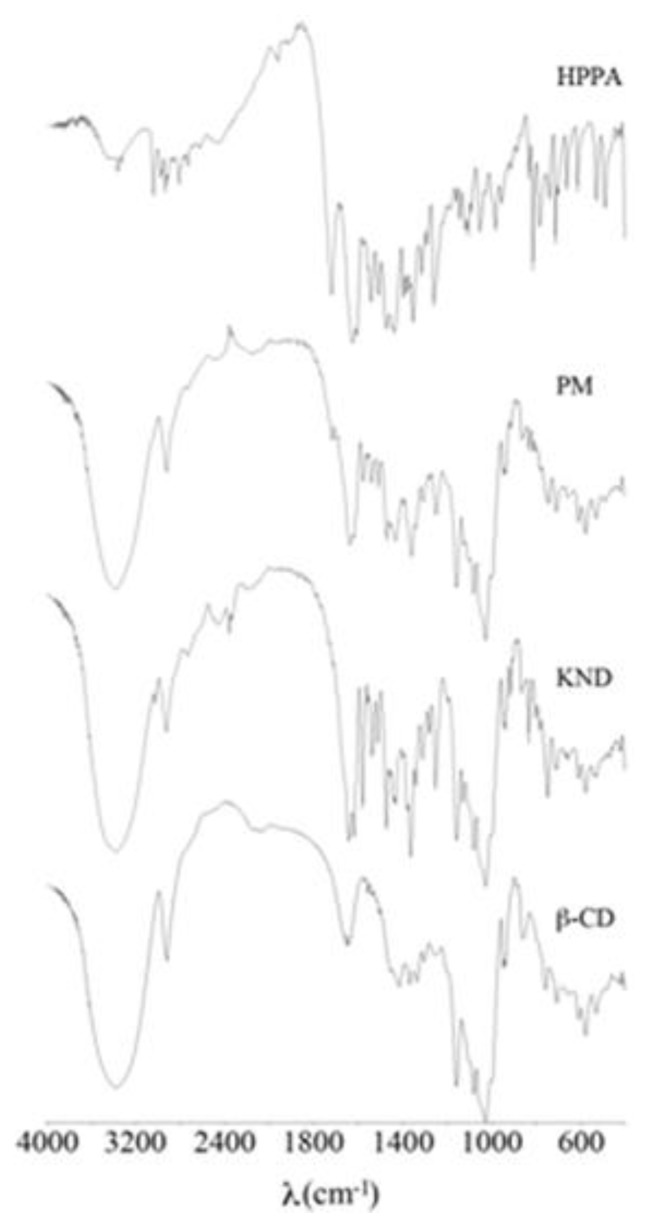
FT-IR spectra of β-CD, KND, PM and HPPA.

The spectra of β-CD, HPPA:β-CD obtained by kneading techniques (KND), physical mixing product (PM) and HPPA are shown in Figure 3. The FT-IR spectrum of β-CD showed prominent absorption bands at 3392 cm^−1^ (for O–H stretching vibrations), 2925 cm^−1^ (for C–H stretching vibrations), 1158 cm^−1^ (for C–O stretching vibrations), and 1028 cm^−1^ (C–O–C stretching vibrations), according to Menezes *et al*. [17]. In the FT-IR spectrum of HPPA, one prominent characteristic peak was found at 3046 cm^−1^, which was assigned to stretching vibration of the –OH group and intramolecular hydrogen bonding. This band also suggested how the –NH (3366 cm^−1^) stretching vibration of the imino-moiety of piperazinyl groups was less prominent due to intense –OH stretching vibration. The band at 1721 cm^−1^ represented the carbonyl C=O stretching. The peak at 1627 cm^−1^ was assigned to the N–H bending vibration of quinolones [18]. The band at 1440 cm^−1^, representing O–C–O stretching vibration of acid, and that at 1261 cm^−1^ suggesting bending vibration of O–H group, indicate the presence of carboxylic acid. The peak at 1354 cm^−1^ was assigned to the out-of-plane bending of the hydroxyl function of the carboxylic acid. The spectrum of the PM product shows approximate superimposition of the individual patterns of both β-CD and HPPA. In the spectrum of the KND, the –NH stretching region of HPPA (3366 cm^−1^) is covered by the O–H stretching band (3392 cm^−1^) of β-CD. The strong carboxyl carbonyl stretching vibration peak at 1721 cm^−1^ of HPPA disappears, the bending vibration of O–H group of carboxylic acid at 1261 cm^−1^ of HPPA shifts to 1252 cm^−1^ with broadening in the complex, indicating the dissociation of the intermolecular hydrogen bonding and interaction through hydrogen bonding with β-CD. The intensification and the shift of the peak at 1354 cm^−1^ of HPPA take place in the complex supporting the involvement of the carboxylic acid function in the complex formation. The intermolecular C–O–C stretch (ether linkage of β-CD) at 1028 cm^−1^ is not affected indicating that the penetration of HPPA is through the axial line of the β-CD cavity [19].

### 2.2. NMR Spectroscopy

**Figure 4:**
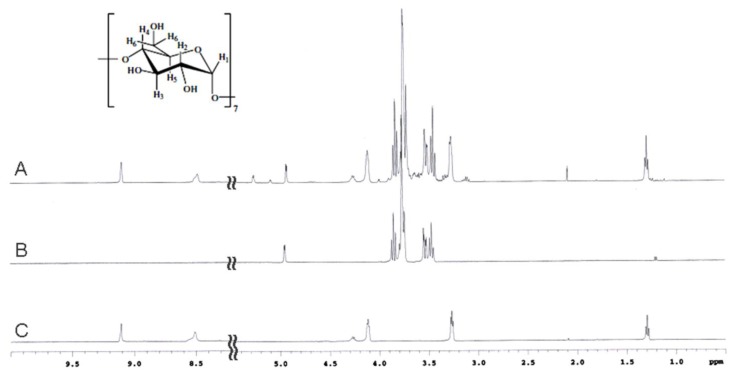
The ^1^H NMR spectra of HPPA:β-CD kneading product (**A**), β-CD (**B**) and HPPA (**C**).

**Figure 5:**
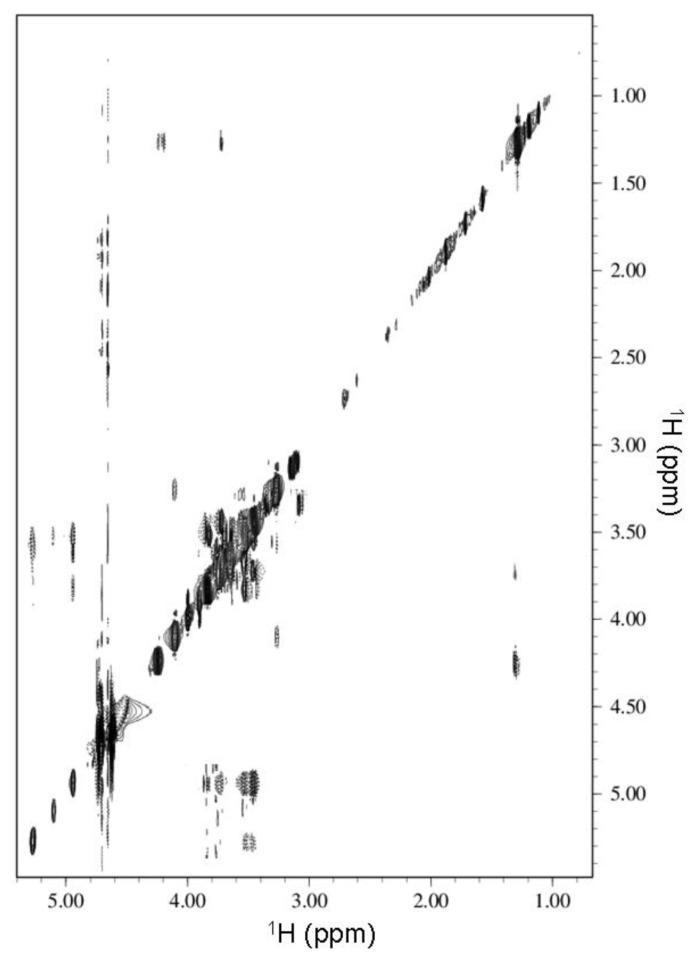
Region of the ^1^H-^1^H ROESY spectrum of the KND product with the negative cross peaks marked with a dotted line.

**Figure 6:**
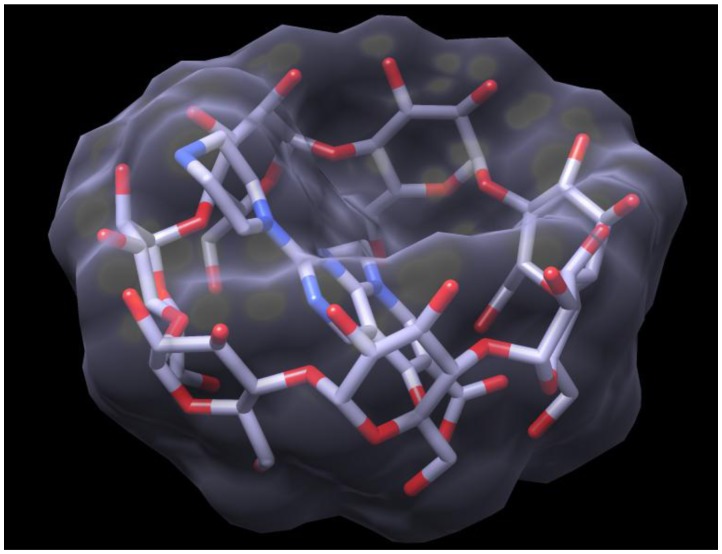
Optimized structure of β-CD in complex with HPPA.

The proton chemical shifts for free HPPA (Figure 1) are summarized in the experimental section. A strong NOE correlation of the proton in position 7 with the proton in position 8a and a weaker NOE between 7 and 8b permitted a discrimination between proton in position 7 and 4. The signals belonging to protons 2a-2a′ and 2b-2b′ were assigned based on the chemical shift reported by Jinxia Li *et al*. [20]. Figure 4 illustrates the ^1^H NMR spectra of β-CD, HPPA and of the KND. The clear changes in the signal pattern for β-CD further confirm the formation of the inclusion complex. The β-CD glucopyranosyl residues in the spectrum of the KND produce three distinct signals for the protons in position 1. Moreover, differences are also evident in the chemical shift of protons in position 3 and 5 localized within the cyclodextrin cavity. This behavior clearly demonstrates the formation of an inclusion complex: the aromatic nature of the guest explains the differences in the magnetic field experienced by the same protons located on different unities. Moreover, changes in the chemical shifts of the HPPA protons in position 4, 7 and 8 (Figure 1) likely indicate that the aromatic part of the molecule is included in the β-CD cavity while the part containing protons in position 2 (the piperazinyl group), whose chemical shift remains almost unperturbed, is likely to be located outside the cavity. This behavior is further confirmed by the ROESY spectrum (Figure 5) in which two unambiguous NOEs prove the proximity of the methyl group of HPPA to the β-CD proton in position 5 and of protons 2b and 2b′ of the piperazinyl group to the β-CD proton in position 2. These data have been confirmed by docking studies (see section 2.3 and Figure 6).

### 2.3. Docking of HPPA onto β-CD

For a deeper understanding of the molecular encapsulation capacity of β-CD, a docking study was performed. The optimized structure of the HPPA: β-CD complex is reported in Figure 6. The results show that the amino ring of HPPA is located inside the hydrophobic cavity of the β-CD. Moreover, in accord with the NMR data, the structure of the complex indicates that the less polar part of the molecule is inserted into the cavity, while the more polar groups are exposed to the bulk solvent outside the opening of the cavity.

### 2.4. UV-Vis Spectroscopy

**Figure 7:**
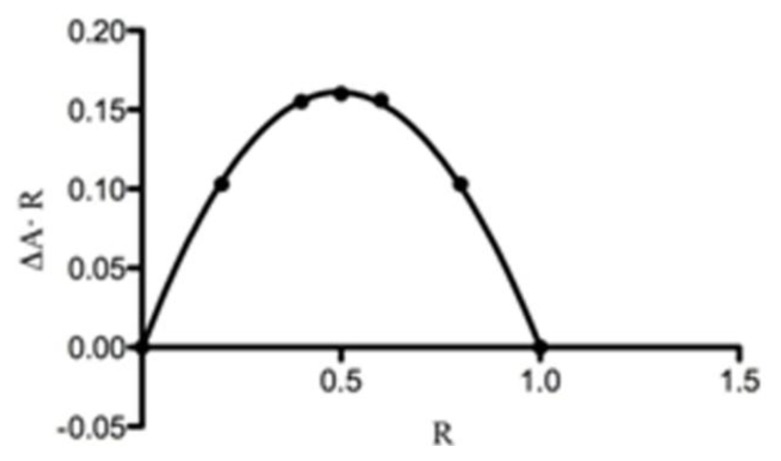
Job plot for the complex HPPA:β-CD (λ = 330.0 nm).

**Figure 8:**
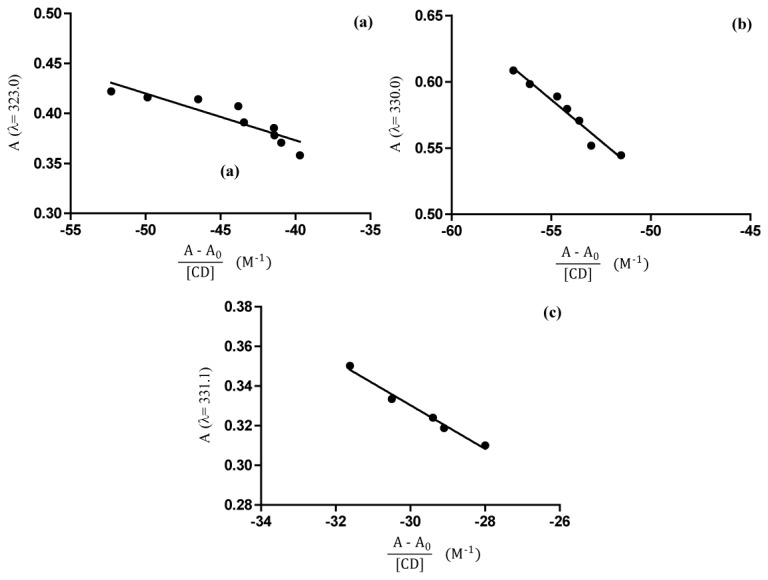
Dependence of HPPA absorbance from β-CD concentration in aqueous solutions with different pH values: (**a**) pH = 4.6 (λ = 323.0); (**b**) pH = 6.8 (λ= 330.0); (**c**) pH = 8.6 (λ = 331.5).

The modification in UV-Vis spectrum of the guest molecule is assumed to result from changes in its solvent microenvironment upon inclusion [21]. The stoichiometry of the complex was determined using Job method [22,23]. The 1:1 stoichiometry is given by the curve maximum at *R* = 0.5 (Figure 7). The evaluation of stability constants by direct spectroscopic methods relies on analytical differences between the free and complexed drug [24]. The HPPA can be present in different cationic, zwitterionic or anionic forms depending on the pH of the solution and each of them may form complexes with the β-CD. Thus, the inclusion of HPPA with β-CD was studied in unbuffered (pH = 6.8), sodium acetate buffered (pH = 4.6) and Tris HCl buffered (pH = 8.6) solutions. The results of the dependence of HPPA absorbance on β-CD concentration are shown in Figure 8. The maximum absorption wavelength of HPPA was pH dependent, being 323.0 nm at pH 4.6, 330.0 nm at pH 6.8, and 331.5 nm at pH 8.6. These results suggest that the inclusion complex was formed between β-CD and HPPA. The *K*_b_ can be obtained from absorbance data using the modified Benesi-Hildebrand [25,26] Equation (1):

(1) $$A=-\frac{1}{K_{\text{b}}}\frac{A-A_{0}}{\left[H\right]}+A_{0}+\Delta ɛ\;[G]$$

where *A* and *A*_0_ are the absorbance of HPPA in the presence and absence of β-CD, respectively, *K*_b_ is the stability constant, [*H*] and [*G*] are the concentrations of β-CD and HPPA, respectively and Δɛ is the difference in the molar absorptivities between free and complexed guest. Therefore, a plot of A versus (*A*−*A*_0_)/[*H*], should give a straight line with slope −1/*K*_b_. The calculated stability constants at different pH were listed in Table 1, from which the formation constant values were very sensitive to pH: *K*_b4.8_ > *K*_b6.8_ ≈ *K*_b8.6_. Thus, it can be concluded that the inclusion of HPPA molecule with β-CD is more suitable in acidic media. The negatively charged HPPA with more hydrophilic character was predominant in basic media, or in aqueous solution, leading to the weaker interaction with β-CD.

#### Phase Solubility Studies

**Figure 9:**
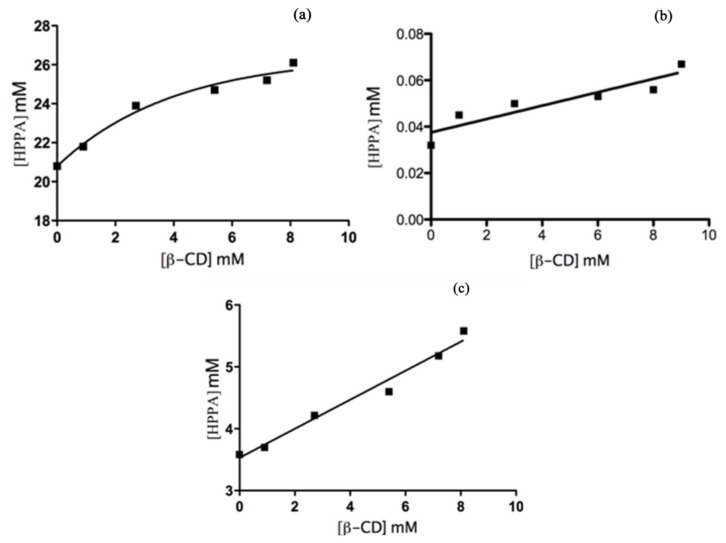
The PSD for the inclusion complex HPPA:β-CD in aqueous solutions with different pH values: (**a**) pH = 4.6; (**b**) pH = 6.8; (**c**) pH = 8.6.

To estimate the stoichiometric ratios and stability constant of the HPPA:β-CD in solution we carried out phase solubility studies measuring the change of solubility of the guest substance as a function of the host concentration. The PSDs for the formation of complexes between HPPA and β-CD at different pH values are shown in Figure 9. The PSD obtained for the HPPA acid form (pH = 4.6) can be classified as Bs type according to Higuchi and Connors [27]. In this case, the HPPA solubility is enhanced by the presence of the host; in particular, a linear increase of solubility for HPPA was observed up to 3 × 10^−3^ M of β-CD. The ascending portion of the Bs type curve indicates that the stoichiometry of the complex is 1:1; then a short plateau region indicates the formation of an insoluble, or with different stoichiometry, complex in the solution at high concentrations of β-CD. Rigorous nonlinear regression [28] of experimental data was conducted to obtain estimates of stability constant. Data analysis and nonlinear regression curve fitting were performed using Prism 5 software (GraphPad, San Diego, CA, USA). The value of Kb was found to be 250.8 M^−1^, according to the value obtained by UV-Vis method. The PSD for HPPA:β-CD in buffered solutions at pH = 8.6 and in unbuffered solution (pH = 6.8), shows that the aqueous solubility of the drug increases linearly as a function of β-CD concentration. The PSD can be classified as the A_L_ type in both solutions and it indicates that the stoichiometry of the complex is 1:1. *K*_b_ values were estimated according to the Equation (2) where *S*_0_, HPPA concentration in the absence of β-CD, was obtained as the y-intercept.

(2) $$K_{\text{b}}=\frac{\text{Slope}}{S_{0}(1-\text{Slope})}$$

The binding constants (Table 1) were estimated to be equal to 88.5 M^−1^ at pH = 6.8 and 87.0 M^−1^ at pH = 8.6; these *K*_b_ values are in agreement with those obtained by UV-Vis. The stability constants obtained for HPPA:β-CD fall in the ideal range between 100 and 1000 M^−1^: smaller values indicated weak interactions between guest and β-CD, while a large value indicates incomplete guest release from the inclusion complex [29]. If the complex is too weak, there is a slight improvement of the water solubility of the host. Moreover, if the complex is too strong, as shown through a stability constant greater than 1000 M^−1^, the complex cannot dissociate easily. In this context, the bioavailability of HPPA is improved by complexation with β-CD. For this reason we have performed a number of bioactivity tests. The complex formation between HPPA and γ-CD was reported by Duran-Meras *et al.* [30] and the values of stability constants obtained by spectrofluorimetric methods resulted in very small values compared to the values obtained for the HPPA:β-CD, probably due to the different size of the γ-CD cavity.

### 2.5. Bioactivity Evaluation

#### 2.5.1. Microbial Susceptibility Test

**Figure 10:**
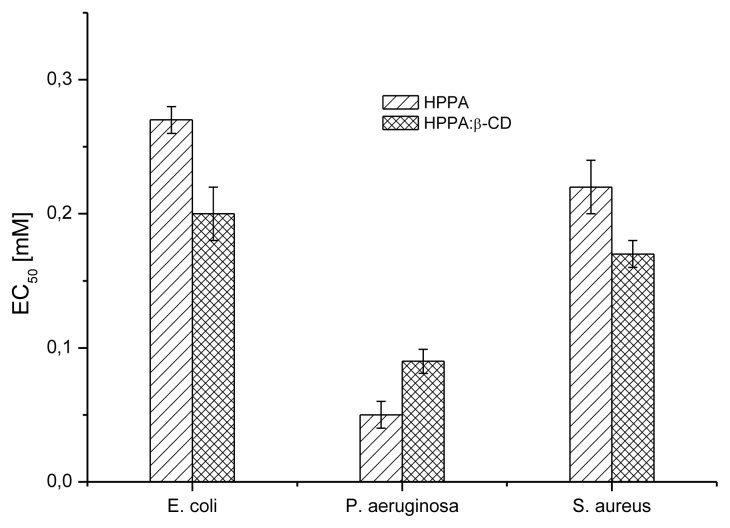
EC_50_ values calculated from the bacterial inhibition percentages for *E. coli*, *P. aeruginosa* and *S. aureus* co-incubated with HPPA and HPPA:β-CD. Error bars show standard deviation.

**Figure 11:**
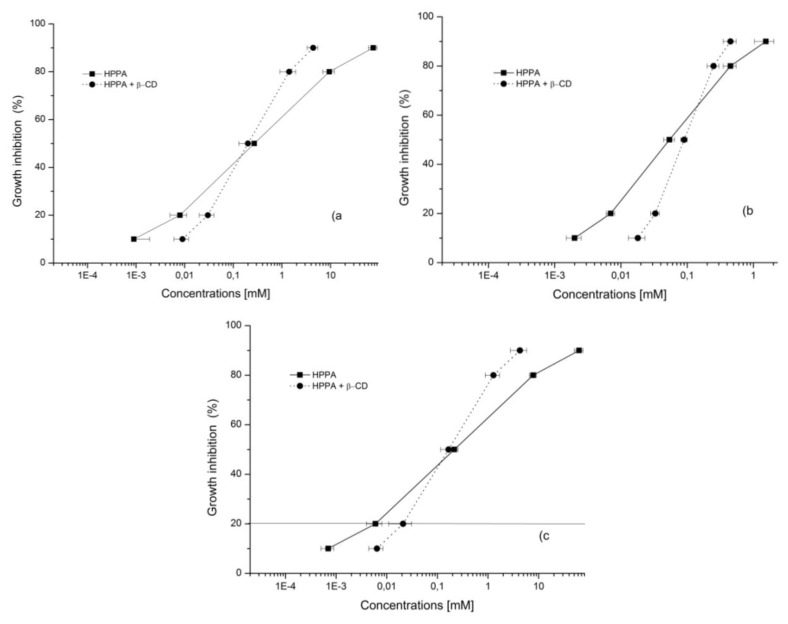
Concentration/response curves of HPPA and HPPA:β-CD showing the EC_90_ values against (**a**) *E. coli*, (**b**) *P. aeruginosa and* (**c**) *S. aureus.*

The antibacterial activities of the pure drug and its complex were tested against two Gram (−), *E. coli* and *P. aeruginosa*, and one Gram (+), *S. aureus*. The effective concentrations inhibiting 50% of bacterial growth (EC_50s_) were determined and results are reported in Figure 10. According to the graph, pipemidic acid appeared to be most effective on *P. aeruginosa* with an EC_50_ value of 0.05 mM. A lower effect was found on *S. aureus* followed by *E. coli* with statistically significant differences in their activity for *p* < 0.05. Furthermore, the efficiency of the complex was tested and results showed a significantly higher antibacterial potential of HPPA:β-CD when compared to HPPA in *E. coli* (*p* < 0.01) and *S. aureus* (*p* < 0.05) by Tukey test. Probably the complex, enhancing HPPA transport through membranes, can improve inhibiting DNA replication HPPA efficacy in *E. coli* and *S. aureus*. Further experiments are needed in order to clarify the possible mechanism of action of HPPA:β-CD in *P. aeruginosa* where the complex activity was lower than that shown by pure compound. In addition to the determination of EC_50_ values, EC_90_ values were also determined. EC_90_ represents the concentration able to generate 90% of bacterial growth inhibition, and it could be preferred to the Minimal Inhibitory Concentration (MIC) even if that is the most frequently used measure to detect the antibacterial activity. MIC, defined as the lowest concentration of the antimicrobial agent that prevents visible growth of a microorganism, is strongly influenced by the experimental conditions to obtain accurate and reproducible results as it is visually determined and based on operator decisions for the chosen concentrations and their spacing. The EC_90_ is an accurate measure because it represents the statistically determined concentration of the antimicrobial agent achieving 90% of growth inhibition compared to the control (without compounds). Results of the growth curves showing EC_90_ inhibition are reported in Figure 11. HPPA:β-CD showed the lowest EC_90_ equal to 0.45 mM against 1.54 mM of HPPA in *P. aeruginosa*. It is interesting to note that the complex improved the efficacy of the HPPA also in *S. aureus* and *E. coli* with EC_90_ values of 4.26 and 4.4 mM, respectively. HPPA showed EC_90_ values equal to 75.7 for *E. coli* and 63.01 mM for *S. aureus*; this means that a lower HPPA amount in the complex (15-fold for *S. aureus* and 17-fold for *E. coli*) would be requested. All concentration/response curves followed the same trend with an increase in inhibition of HPPA:β-CD compared to HPPA at increasing concentrations. These findings could be of great interest in urinary tract infection therapy as HPPA is used, against *E. coli* and *P. aeruginosa*, in these types of infections [31–33]. The inclusion complex of HPPA with β-CD would allow lower doses of HPPA in therapy and consequently, lower adverse effects. HPPA has been extensively studied for its antibacterial potential especially using the MIC value determination, but results vary widely because of different test conditions and of non-standardized procedures. Our results partially agree with Yang *et al.* [34] who reported high inhibitory action against *E. coli*, slight inhibition against *P. aeruginosa* but no sensitivity on *S. aureus*. On the other hand, Efthimiadou *et al.* [8] found the highest antibacterial activity for HPPA on *S. aureus*. No studies were found using antibacterial assays on HPPA included into β-CD.

#### 2.5.2. MTT Assay

**Figure 12:**
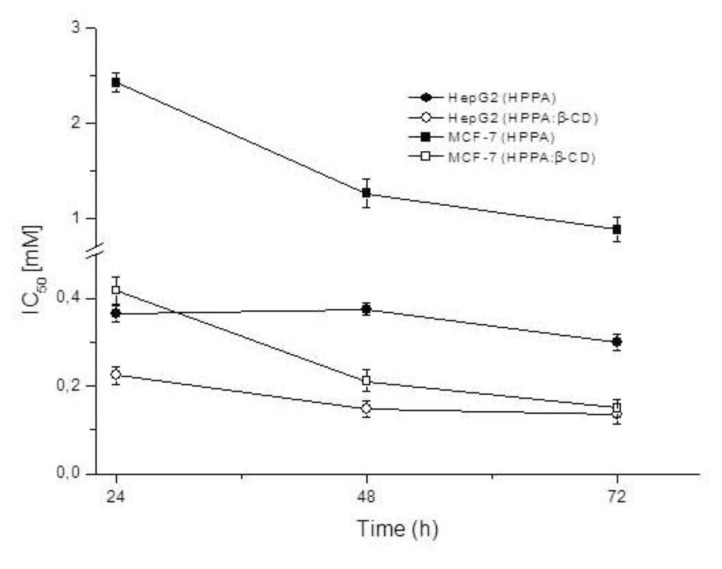
Antitumor activity expressed as IC_50_ (mM) against MCF-7 and HepG2 cells of HPPA and HPPA:β-CD after 24, 48 and 72 h of co-incubation.

MTT assays determine the citotoxicity of compounds considering the number of viable cells. In this study, human hepatocellular carcinoma (HepG2) and human breast adenocarcinoma (MCF-7) cell lines were used to detect possible differences in antitumor activity between HPPA and its complex with β-CD. The results are reported in Figure 12 where IC_50_ values are plotted against different incubation times. Our findings show that HPPA has a higher antitumor activity, around one order of magnitude, against hepatocellular carcinoma cells compared to breast cancer cells. The highest efficacy was shown at 72 h both for MCF-7 and HepG2. It is worth to underline that significant differences (*p* < 0.01) were found between IC_50_ values only at 24 and 48 h for HepG2 while IC_50s_ for MCF-7 were statistically different for *p* < 0.01 both at 24 and 48 h, and 24 and 72 h; a significativity of 0.05 was found between 48 and 72 h. The efficiencies of HPPA:β-CD compared to the pure compound evidenced that the inhibition of proliferation in MCF-7 cells was remarkable with IC_50_ values decreasing from 0.88 to 0.15 mM. The activity of the complex on the HepG2 was lower but, in any case, HPPA:β-CD showed an IC_50_ value more than half the value found for HPPA alone (0.14 *versus* 0.3 mM). At each time test the comparison between the complex and the pure compound showed significant differences for *p* < 0.01. Both for MCF-7 and HepG2, the differences in activity can be reasonably explained by the modified chemistry of the complex. In fact, the inclusion of pipemidic acid into β-CD can increase the transport of HPPA through the cellular membranes improving its cytotoxic activity. The quinolone investigated has been previously studied for its antitumor activity by other researchers who found changes in its potential when in complex with polyoxometallates (POMs) whose application in medicine is difficult for their low hydrolytic stability and low selectivity as well as high toxicity [35,36]. In the present study, the use of cyclodextrin allows the formation of a safe complex that increases the aqueous solubility of the drug, its bioavailability and then its antitumor activity.

## 3. Experimental Section

### 3.1. Materials

β-CD and HPPA were purchased from Sigma-Aldrich. The buffer solutions at different pHs were prepared by adding the appropriate amounts of sodium acetate and Tris HCl; the solutions were prepared just before taking each measurement. All the reagents and solvents were of analytical grade. Double distilled and MilliQ water was used throughout the experiments.

### 3.2. Preparation of Solid Binary System

The HPPA:β-CD solid binary system was prepared in 1:1 molar ratio by following different methods.

#### 3.2.1. Physical Mixing Method

The Physical Mixing product (PM) was prepared by simply blending powders of β-CD (0.100 g) and HPPA (0.027 g) in a mortar for 5 min, at room temperature.

#### 3.2.2. Kneading Method

Kneading product (KND) was obtained by adding a small volume of a water-methanol (50/50, *v*/*v*) solution to the HPPA (0.040 g) and β-CD (0.150 g) physical mixture and kneading the resultant mixture thoroughly with a pestle to obtain a homogeneous paste until the solvent was completely removed. The sample was dried at 40 °C in oven for 30 min to remove traces of solvent. The dried mass was pulverized.

### 3.3. Fourier Transform Infrared (FT-IR) Spectroscopy

FT-IR analysis was performed on Perkin Elmer Spectrum GX spectrometer (Waltham, MA, USA). FT-IR measurements of the pure materials (HPPA and β-CD), binary system and PM were carried out using the KBr disks method. The KBr disks were prepared by compressing the powder. The scanning range was kept from 4000 to 400 cm^−1^, with a resolution of 1 cm^−1^.

### 3.4. Nuclear Magnetic Resonance (NMR) Spectroscopy

NMR spectra of β-CD, HPPA and their kneading product HPPA:β-CD (KND) were carried out at 500 MHz using a Varian UNITY 500 spectrometer. ^2^H_2_O (99.9% relative isotopic abundance) was purchased from Cambridge Isotope Laboratories. The proton chemical shifts were collected at 298 K, at pH = 5.8, and referenced to external TMS (δ = 0 ppm). Two-dimensional phase-sensitive TOCSY, NOESY, ROESY spectra [37] were collected using the States and Haberkorn method. Squared-shifted sine-bell functions were applied in both dimensions before Fourier transformation and baseline correction. TOCSY, NOESY and ROESY experiments were recorded with mixing times of 70, 200 and 150 ms, respectively. Water suppression, when necessary, was achieved using the DPFGSE sequence [38]. The data were processed and analyzed using the VNMRJ and CARA software [39]. The proton chemical shifts for free HPPA (Figure 1) are summarized: 4.11 ppm (proton in position 2a and 2a′), 3.28 ppm (2b and 2b′), 8.51 ppm (7), 9.11 ppm (4), 4.27 ppm (8a), 1.30 ppm (8b).

### 3.5. Molecular Docking

Docking was performed using the Hex software [40] version 6.3. The PDB files of CD and HPPA were uploaded as inputs into Hex and treated as receptor and ligand, respectively. All the input files were analyzed using the spherical harmonic surface of the Hex. Computations were performed by using the shape complementary scoring function, with 16 and 30 expansion orders for the initial and final search steps. The full list of parameters is given in the Table 2. Structure refinement and energy minimization were performed with Hex itself. Based on the energy minimization the best pose of the docked complex was selected.

### 3.6. Ultraviolet-Visible (UV-Vis) Spectroscopy

For all UV-Vis spectroscopy studies, a UV-1700 Spectrometer (Shimadzu, Tokyo, Japan) was used with 1 cm matched quartz cuvettes. All measurements were recorded in the wavelength range 200–400 nm at room temperature. The stoichiometry of the complex was determined using the continuous variation method [22,23]. According to this method, 0.05 mM unbuffered solutions of HPPA and β-CD were mixed at different concentration ratios *R* = [β-CD]/([HPPA] + [β-CD]) keeping the volume constant. The stoichiometric ratio was obtained by plotting Δ*A* × *R* against *R* (where Δ*A* is the difference of absorbance of HPPA without and with β-CD) and finding the *R* value corresponding to the extreme of this dependence. The evaluation of *K*_b_ by direct spectroscopic methods relies on analytical differences between the free and complexed drug [24]. Changes in the absorption intensity of HPPA at 330 nm (pH = 6.8), at 323 nm (pH = 4.6), and at 331.5 nm (pH = 8.6), were monitored as a function of β-CD concentration to measure the Kb. The HPPA concentration was kept constant (0.05 mM) while β-CD concentration was varied (0–1.85 mM). The absorbances of the resulting solutions were measured at different pH values obtained, adding proper buffer solutions. All absorption measurements were made against a blank solution treated the same way, but without HPPA and β-CD. Measurements of pH were performed using a calibrated CRISON pH-meter Basic 20. To conveniently calculate the *K*_b_, we needed to rearrange the Benesi-Hildebrand equation [25] into a straight line form [26] shown in the Equation (1).

#### Phase Solubility Studies

Phase solubility studies were performed according to the method reported by Higuchi and Connors [27]. HPPA, in an amount (130.5 mg) that exceeded its solubility, was added into vials in which there were various concentrations of β-CD (0–9 mM) and unbuffered MilliQ water (5 mL) at pH = 6.8, or buffered MilliQ water at pH = 4.6 or pH = 8.6. The vials were then sealed and thermostatically shaken at 40 °C for 100 h. This amount of time is considered sufficient to reach equilibrium [10]. Subsequently, using a syringe the aliquots were filtered immediately through a 0.45 μm Millipore membrane filter. A portion of the sample was analyzed by UV spectrophotometer at different λ_max_: 323 nm (pH = 4.6); 330 nm (pH = 6.8); 331.5 nm (pH = 8.6). These wavelengths for the HPPA specific molar absorbance were obtained from the construction of calibration curves. The solubility experiments were performed in triplicate. The total concentration of HPPA solubilized was calculated as: [HPPA] = *A*_HPPA_/ɛ_HPPA_ where *A*_HPPA_ is the phase solubility test absorbance and ɛ_HPPA_ is the specific molar absorbance of HPPA. It is implicitly assumed in the Higuchi and Connors [27] procedure that the ɛHPPA value does not change upon complexation with β-CD [41]. Phase Solubility Diagrams (PSD) were represented as the total dissolved drug concentration against the concentration of β-CD. The Kb for each complex was calculated from the slope of the straight-line portion curve, when PSD is *A*_L_ type according to the Equation 2. The Kb for each complex, instead, was calculated from the rigorous nonlinear regression curve fitting [28] performed using Prism 5 software (GraphPad, San Diego, CA, USA), when PSD results Bs type.

### 3.7. Bioactivity Evaluation

#### 3.7.1. Microbial Susceptibility Test

Two gram-negative bacteria and one gram-positive bacterium were tested in the Broth-based turbidometric assay. Among the former, *P. aeruginosa* and *E. coli* ATCC13762 were chosen. *P. aeruginosa* is a free-living bacterium commonly found in soil and water while *E. coli* is an opportunistic pathogen of clinical relevance for humans. The gram-positive bacterium utilized was the pathogen *S. aureus* ATCC6538. All bacteria were stored frozen in 90% (*v*/*v*) glycerol in Tryptic Soy Broth (TSB, Oxoid) at −80 °C until use when they were cultured in TSB overnight at 37 °C. The test was performed using 96-well flat bottom micro-titre plates (Sarstedt, Italy) aseptically prepared. TSB broth aliquots (100 μL) were poured into each well and 100 μL of HPPA or of its complex were added to the top row and serial two-fold dilutions made by transferring 100 μL each time to obtain the desired concentrations. The concentrations of HPPA were in the range from 0.013 to 3.376 mM, while in the complex, HPPA concentrations were from 0.003 up to 0.712 mM. Then, each well, except the TSB control (only medium) row, was inoculated with 100 μL of the bacterial culture corresponding to 1 × 10^4^ CFU [42]. The final volume in each well was 200 μL. Before incubation at 37 °C for 20–24 h, the plates were covered by a sterile film against evaporation. The optical density of each well at 620 nm was recorded using a microplate reader (Spectra Fluor, Tecan, Mannedorf, Switzeland). The results were compared to the control containing physiologic solution (0.9% NaCl) in TSB and inoculum. Each experiment was performed in quadruplicate. While testing, a positive control was carried out with streptomycin at a starting concentration of 10 μg/mL. The bacterial inhibition percentage was determined as follows: 100 − (1 − OD_620_ of test sample)/OD620 control bacterial growth × 100. The effective concentrations inhibiting 50% (EC_50_) and 90% (EC_90_) of bacterial growth were determined.

#### 3.7.2. MTT-Assay

The cell growth inhibition on human hepatocellular carcinoma (HepG2) and human breast adenocarcinoma (MCF-7) cell lines was determined by MTT assay, following the procedure of Mothanna *et al.* [43]. In this assay, the increase or decrease in the number of viable cells is linearly connected with the mitochondrial activity, highlighted by the conversion of the tetrazolium salt 3-(4,5-dimethylthiazol-2-yl)-2,5-diphenyltetrazolium bromide (MTT) into formazan crystals, which can be solubilized and spectrophotometrically quantified. The HPPA and HPPA:β-CD activity is expressed as the concentration of the compound able to achieve 50% growth inhibition compared to the growth of the untreated control (50% inhibitory concentration, IC_50_). The routine maintenance of HepG2 and MCF-7 cell lines consisted of their growth in Roswell Park Memorial Institute (RPMI) supplemented with 10% fetal bovine serum (FBS), 2% l-Glutamine, 2% HEPES and 1% penicillin/streptomycin (10000 U/mL) (Lonza, Verviers, Belgium), at 37 °C in an atmosphere of 5% CO_2_ air under saturating humidity. In the MTT assay, HepG2 and MCF-7 cells were plated at 1 × 10^4^ cells/well in a 96-well tissue culture plate and incubated for a sufficient time to assure attachment and 40% to 60% confluence. After 24 h, the medium was aspirated off and replaced with fresh medium (200 μL) containing the samples under investigation. HPPA concentration ranged from 0.05 to 1.65 mM, while in the complex, HPPA concentrations ranged from 0.01 to 0.35 mM. Three plates were incubated at 37 °C, 5% CO_2_, for the OD measurements at 24, 48 and 72 h, respectively. After the respective incubation time, MTT solution (20 μL) was added in each well, then incubated for a further 4 h. After that, MTT-containing medium was gently removed and replaced with 2-propanol (200 μL per well). The plates were read at a microtitre plate reader at 590 nm (Spectrafluor, Tecan, Mannedorf, Switzeland). For each compound tested, the IC_50_ was calculated from the dose-response curves.

#### 3.7.3. Data Analysis

HPPA and HPPA:β-CD were examined three times (three independent assays) and results expressed as nominal concentrations. The results of MTT and turbidometric test were analyzed using Toxcalc™ (Tidepool Scientific Software, McKinleyville CA, USA, 1996). IC_50_ values of the cell growth inhibition in HepG2 and MCF-7 cells as well as EC_50_ and EC_90_ values of bacterial growth were calculated by concentration/response regression using Maximum Likelihood-Logit method.

## 4. Conclusions

An inclusion complex of HPPA with β-CD (HPPA:β-CD) was prepared in the solid state by kneading method and physical mixture. The formation of the inclusion complex was confirmed by FT-IR spectroscopy. In aqueous solution, the effect of β-CD on the absorption spectra of HPPA has been studied in buffered and unbuffered solutions at different pH. The 1:1 stoichiometry was established by a Job plot and confirmed by phase solubility studies. The inclusion complex formation was investigated by UV-Vis titration and the HPPA:β-CD stability constants (*K*_b_), calculated for each pH value, resulted 215.2 M^−1^ at pH = 4.6, 79.7 M^−1^ at pH = 6.8 and 90.7 M^−1^ at pH = 8.6. The phase solubility studies, according to Higuchi and Connors method, were also performed at different pH values. The HPPA solubility is enhanced by the presence of the host in all the solutions. The PSD for HPPA:β-CD in buffer solutions at pH = 4.6 can be classified as Bs type and the Kb was estimated to be equal to 251.0 M^−1^. The PSD for HPPA:β-CD in buffer solutions at pH = 8.6 and in unbuffered solution (pH = 6.8), instead, can be classified as the A_L_ type and, utilizing phase solubility diagrams data, the *K*_b_ were estimated to be equal to 88.5 M^−1^ at pH = 6.8 and 87.0 M^−1^ at pH = 8.6 respectively. The obtained inclusion complex was more stable under the acidic conditions due to the hydrophobic effect. NMR, docking and FT-IR of KND revealed that the less polar part of the molecule is inserted into the cavity, while the more polar groups are exposed to the bulk solvent. The biological activity of HPPA and its complex was determined to detect possible differences in their antibacterial capability. The results, expressed as EC_50_, showed that the complex exerted a robust action against *E. coli* and *S. aureus*. The calculation of EC_90_ values confirmed this result for *E. coli* in terms of HPPA amount reduction, demonstrating that the complex has the potential, after further investigation, to replace the use of HPPA in medicine. Furthermore, these compounds, tested on human breast adenocarcinoma MCF-7 cells and, for the first time to our knowledge, on the human hepatoblastoma HepG2 cell line, exhibited antitumor activity and the complex revealed a higher anticancer effect than HPPA, especially for HepG2 cells, opening interesting scenarios to explore. In conclusion, in this study we demonstrated an improvement of solubility of HPPA using β-CD. Moreover, the results indicate that the bioactivity of the drug remains in the HPPA:β-CD, indicating that CDs may serve as excipient in pharmaceutical formulations. Further detailed studies, including the clarification of the structure-bioactivity relationship of the cyclodextrin inclusion complex by NMR analysis, are now in progress in our laboratories.

## Figures and Tables

**Table 1:** The stability constants of β-CD with HPPA were calculated by absorbance measurement in different pH values.

pH	*K*_b_ (M^−1^) UV-Vis	*K*_b_ (M^−1^) PSD
4.6	215.2	250.8
6.8	79.7	88.5
8.6	90.8	86.7

**Table 2:** Hex parameters used in this study.

Correlation type	Shape only
FFT Mode	3D fast life
Receptor range angle	180
Ligand range angle	180
Twist range	360
Distance range	40
Docking main scan	16
Docking main search	30
